# Supplementary material for: Multiple Antimicrobial Effects of Hybrid Peptides Synthesized Based on the Sequence of Ribosomal S1 Protein from Staphylococcus aureus
Source: Int J Mol Sci. 2022 Jan 4;23(1):524. doi: 10.3390/ijms23010524 (PMC8745237; doi:10.3390/ijms23010524)
Supplement: Supplementary file 1 [file ijms-23-00524-s001.zip › ijms-1494758-supplementary.pdf]

Table S1. Prediction of amyloidogenic regions of the synthetized peptides.

№	Peptide	Sequence	Algorithm and program for predicting amyloidogenic regions			
			**AGGRESCAN** [1]	**FoldAmyloid** [2]	**Waltz** [3]	**MetAmyl** [4]
1	G10G	GVVVRLANFG	1-8 a.a.<br>GVVVRLAN	1-8 a.a.<br>GVVVRLAN	5-10 a.a.<br>RLANFG	1-7 a.a.<br>GVVVRLA
2	Q10I	QQVNVKILGI	5-10 a.a.<br>VKILGI	5-10 a.a.<br>VKILGI	3-10 a.a.<br>VNVKILGI	2-9 a.a.<br>QVNVKILG
3	V10F	VVVHINGGKF	1-5 a.a.<br>VVVHI	Not found	1-10 a.a.<br>VVVHINGGKF	1-8 a.a.<br>VVVHINGG
4	V10I	VQGLVHISEI	3-10 a.a.<br>GLVHISEI	2-9 a.a.<br>QGLVHISE	3-10 a.a.<br>GLVHISEI	1-10 a.a.<br>VQGLVHISEI
5	R23F	RKKRRQRRRGG-Sar*(A)-GVVVHI-Asi**(D)GGKF	12-18 a.a<br>AGVVVHI	Not found	18-23 a.a<br>IDGGKF	10-20 a.a.<br>GGAGVVVHIDG
6	R23F	RKKRRQRRRGG-Sar(P)-GVVVHI-Asi(N)GGKF	13-18 a.a.<br>GVVVHI	Not found	18-23 a.a<br>IDGGKF	11-20 a.a.<br>GPGVVVHIDG
7	R23DI	RKKRRQRRRGG-Sar(A)-GLTQFGAFIDI	18-23 a.a.<br>GAFIDI	18-23 a.a.<br>GAFIDI	11-23 a.a<br>GAGLTQFGAFIDI	Not found
8	R23DI	RKKRRQRRRGG-Sar(P)-GLTQFGAFIDI	18-23 a.a.<br>GAFIDI	18-23 a.a.<br>GAFIDI	16-23 a.a<br>QFGAFIDI	Not found
9	R23EI	RKKRRQRRRGG-Sar(A)-GVQGLVHISEI	15-23 a.a.<br>QGLVHISEI	16-20 a.a.<br>GLVHI	16-23 a.a<br>GLVHISEI	10-23 a.a.<br>GGAGVQGLVHISEI
10	R23EI	RKKRRQRRRGG-Sar(P)-GVQGLVHISEI	15-23 a.a.<br>QGLVHISEI	16-22 a.a.<br>GLVHISE	16-23 a.a<br>GLVHISEI	10-23 a.a.<br>GGPGVQGLVHISEI

* For calculations, sarcosine (Sar) was replaced by analogs similar in properties and structure: alanine (A) and proline (P).
** For calculations, aminosuccinimide (Asi) was replaced by analogs of similar properties and structure: asparagine (N).

Table S2. Prediction of the physicochemical properties of the peptides.

№	Peptide	Sequence	Lenght	**ProtParam** [5] **(ExPASy Server)**					**DBAASP v3.0** [6] **(MF Scale)**	
				**Z**	**Theoretical pI**	**GRAVY**	**The instability index (II)**	**Aliphatic index (AI)**	**μHn**	**Hn**
1	G10G	GVVVRLANFG	10	+1.0	10.5	1.220	-6.03	136	0.82	-1.13
2	Q10I	QQVNVKILGI	10	+1.0	9.82	0.640	20.12	175	0.56	-0.55
3	V10F	VVVHINGGKF	10	+1.0	10.09	0.850	41.76	126	0.29	-0.47
4	V10I	VQGLVHISEI	10	-1.0	5.14	0.980	91.46	175	0.83	-0.91
5	R23F	RKKRRQRRRGG-Sar*(A)-	23	+8.0	12.7	-1.03	124.43	59.13	0.19	0.68

		GVVVHI-Asi**(D)GGKF								
6	R23F	RKKRRQRRRGG-Sar(P)-GVVVHI-Asi(N)GGKF	23	+8.0	12.4	-1.417	129.28	54.78	0.25	0.61
7	R23DI	RKKRRQRRRGG-Sar(A)-GLTQFGAFIDI	23	+7.0	12.3	-1.110	94.56	59.57	0.29	0.23
8	R23DI	RKKRRQRRRGG-Sar(P)-GLTQFGAFIDI	23	+7.0	12.7	-1.260	98.25	55.22	0.25	0.16
9	R23EI	RKKRRQRRRGG-Sar(A)-GVQGLVHISEI	23	+7.0	12.4	-1.213	80.43	140.66	0.30	0.51
10	R23EI	RKKRRQRRRGG-Sar(P)-GVQGLVHISEI	23	+7.0	12.4	-1.361	144.35	76.09	0.25	0.44

* For calculations, sarcosine (Sar) was replaced by analogs similar in properties and structure: alanine (A) and proline (P).
** For calculations, aminosuccinimide (Asi) was replaced by analogs of similar properties and structure: asparagine (N). **Z** is the value of charge at pH 7, **pI** is the theoretical value of isoelectric point, **GRAVY** is the grand average of hydropathicity, **II** is the instability index, **AI** is the aliphatic index, **μHn** is the normalized hydrophobic moment, **Hn** is the normalized hydrophobicity [5,6].

Table S3. Prediction of antimicrobial properties of the peptides.

№	Peptide	CAMP$_{R3}$ [7]						
		SVM		RF		DA		ANN
		Class	AMP Probability	Class	AMP Probability	Class	AMP Probability	Class
1	G10G	NAMP§	0,05	NAMP	0,35	NAMP	0,40	AMP§§
2	Q10I	NAMP	0,11	NAMP	0,44	NAMP	0,43	AMP
3	V10F	NAMP	0,4	NAMP	0,36	AMP	0,86	AMP
4	V10I	NAMP	0,04	NAMP	0,3	NAMP	0,09	NAMP
5	R23F	NAMP	0,02	AMP	0,5	AMP	0,99	AMP
6	R23F	NAMP	0.07	AMP	0.6	AMP	0.98	AMP
7	R23DI	NAMP	0.03	AMP	0.55	AMP	0.99	AMP
8	R23DI	NAMP	0.06	AMP	0.57	AMP	0.99	AMP
9	R23EI	NAMP	0.02	AMP	0.51	AMP	0.91	AMP
10	R23EI	NAMP	0.07	NAMP	0.5	AMP	0.87	NAMP

§ It is predicted as a peptide that does not exhibit antimicrobial activity. The prediction level is less than 0.5. §§ It is predicted as a peptide with antimicrobial activity. The prediction level is greater than 0.5. Antimicrobial activity was predicted using algorithms: **SVM** is a "support vector machine", **RF** is a "random forest", **DA** is a "discriminant analysis", **ANN** is an "artificial neural network" [7].

Table S4. Results of preliminary testing the antimicrobial properties of the VVVHINGGKF (V10F), GVVVRLANFG (G10G), VQGLVHISEI (V10I), QQVNVKILGI (Q10I) peptides against *S. aureus*, MRSA, *B. cereus*, and *E. coli* bacteria cells on agar.

	1 μM	10 μM	100 μM	1000 μM	10000 μM
**VVVHINGGKF (V10F)**					
S. aureus (209P strain)	Non-AMP	Non-AMP	Non-AMP	Non-AMP	Non-AMP
MRSA (ATCC 43300 strain)	Non-AMP	Non-AMP	Non-AMP	Non-AMP	Non-AMP

MRSA (SA 180-F strain)	Non-AMP	Non-AMP	Non-AMP	Non-AMP	**AMP**
E. coli (K12 strain)	Non-AMP	Non-AMP	Non-AMP	Non-AMP	Non-AMP
B. cereus (IP-5812 strain)	Non-AMP	Non-AMP	Non-AMP	Non-AMP	Non-AMP
**GVVVRLANFG (G10G)**					
S. aureus (209P strain)	Non-AMP	Non-AMP	Non-AMP	Non-AMP	**AMP**
MRSA (ATCC 43300 strain)	Non-AMP	Non-AMP	Non-AMP	Non-AMP	Non-AMP
MRSA (SA 180-F strain)	Non-AMP	Non-AMP	Non-AMP	Non-AMP	Non-AMP
E. coli (K12 strain)	Non-AMP	Non-AMP	Non-AMP	Non-AMP	Non-AMP
B. cereus (IP-5812 strain)	Non-AMP	Non-AMP	Non-AMP	Non-AMP	Non-AMP
**VQGLVHISEI (V10I)**					
S. aureus (209P strain)	Non-AMP	Non-AMP	Non-AMP	Non-AMP	Non-AMP
MRSA (ATCC 43300 strain)	Non-AMP	Non-AMP	Non-AMP	Non-AMP	Non-AMP
MRSA (SA 180-F strain)	Non-AMP	Non-AMP	Non-AMP	Non-AMP	**AMP**
E. coli (K12 strain)	Non-AMP	Non-AMP	Non-AMP	Non-AMP	Non-AMP
B. cereus (IP-5812 strain)	Non-AMP	Non-AMP	Non-AMP	Non-AMP	Non-AMP
**QQVNVKILGI (Q10I)**					
S. aureus (209P strain)	Non-AMP	Non-AMP	Non-AMP	Non-AMP	Non-AMP
MRSA (ATCC 43300 strain)	Non-AMP	Non-AMP	Non-AMP	Non-AMP	Non-AMP
MRSA (SA 180-F strain)	Non-AMP	Non-AMP	Non-AMP	Non-AMP	Non-AMP
E. coli (K12 strain)	Non-AMP	Non-AMP	Non-AMP	Non-AMP	Non-AMP
B. cereus (IP-5812 strain)	Non-AMP	Non-AMP	Non-AMP	Non-AMP	Non-AMP

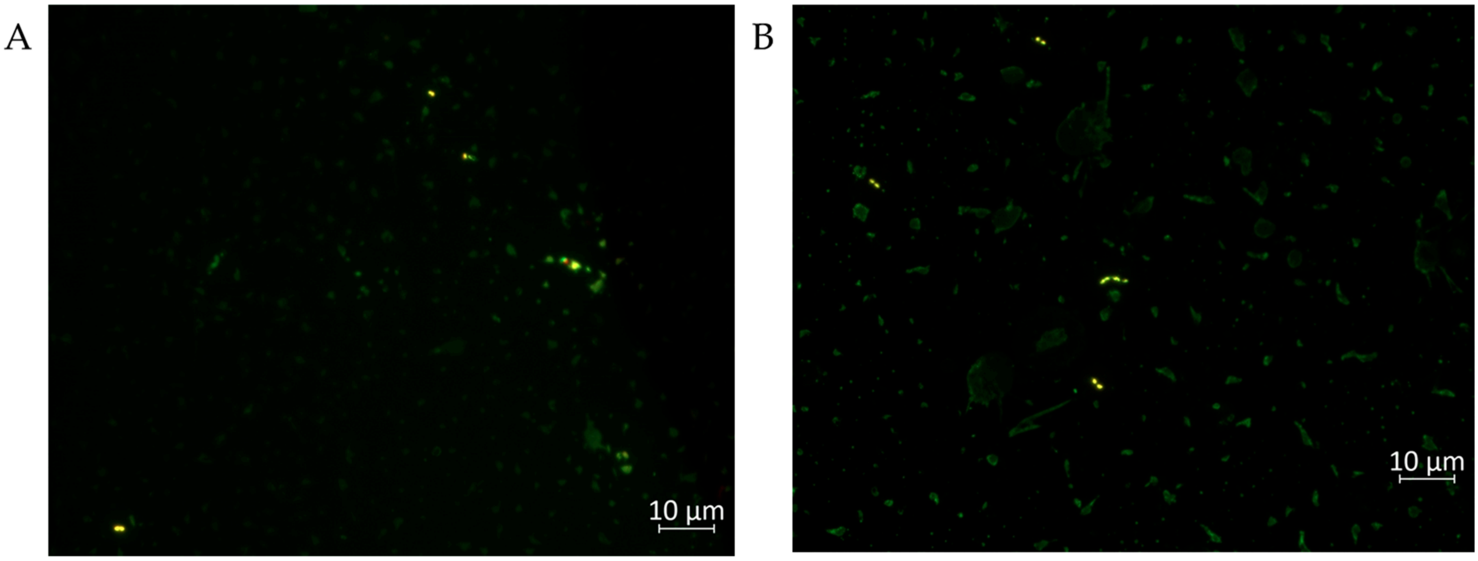

Figure S1. Fluorescence microscopic images of (**A**) MRSA cells (ATCC 43300 strain) and (**B**) *S. aureus* cells (209P strain) after one day of co-incubation with gentamicin sulfate concentration of 1700 µM. Bacterial cells stained with SYTO 9 (green) and propidium iodide (red). Green color indicates the living cells, yellow color, turning into red, indicates a violation of the vital functions of cells.

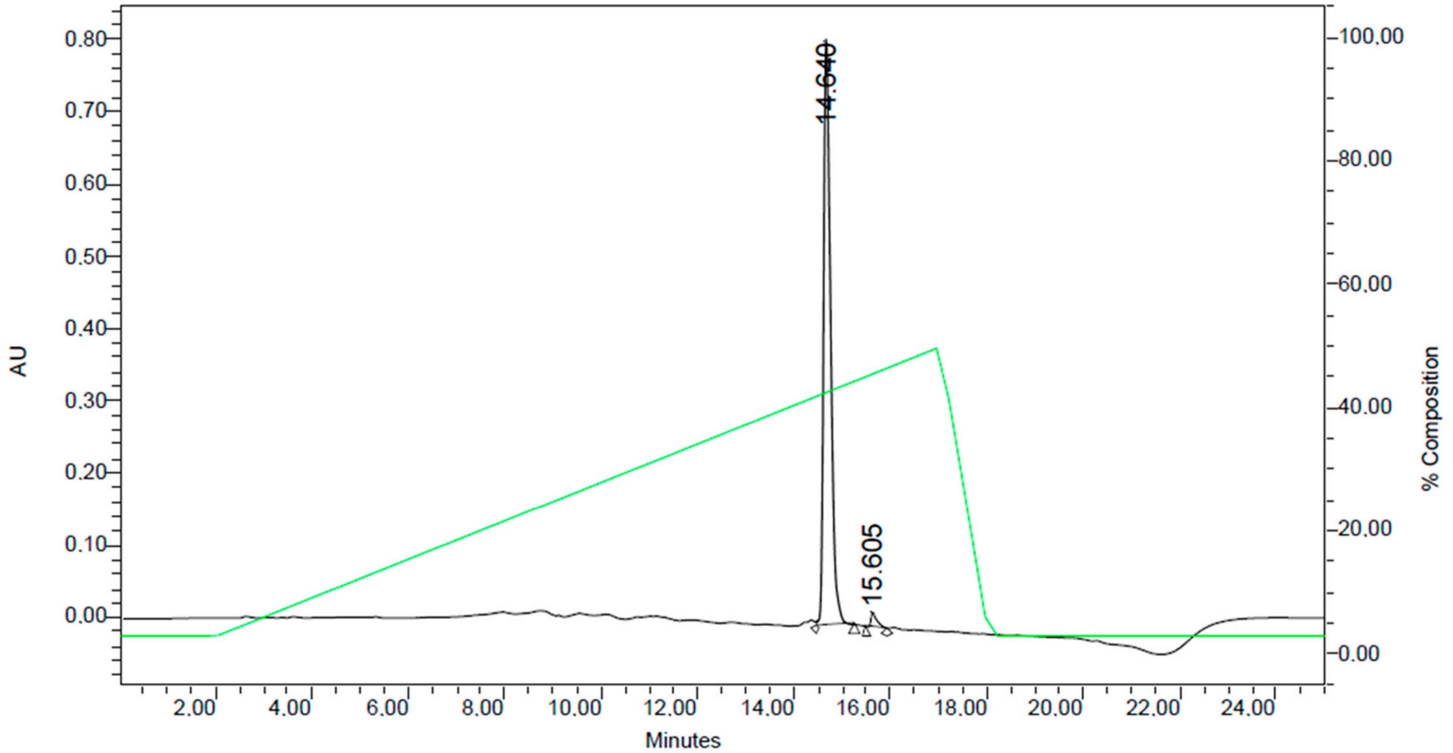

Figure S2. The chromatogram of sample R23F peptide (run time for peak of R23F was 14.640 min).

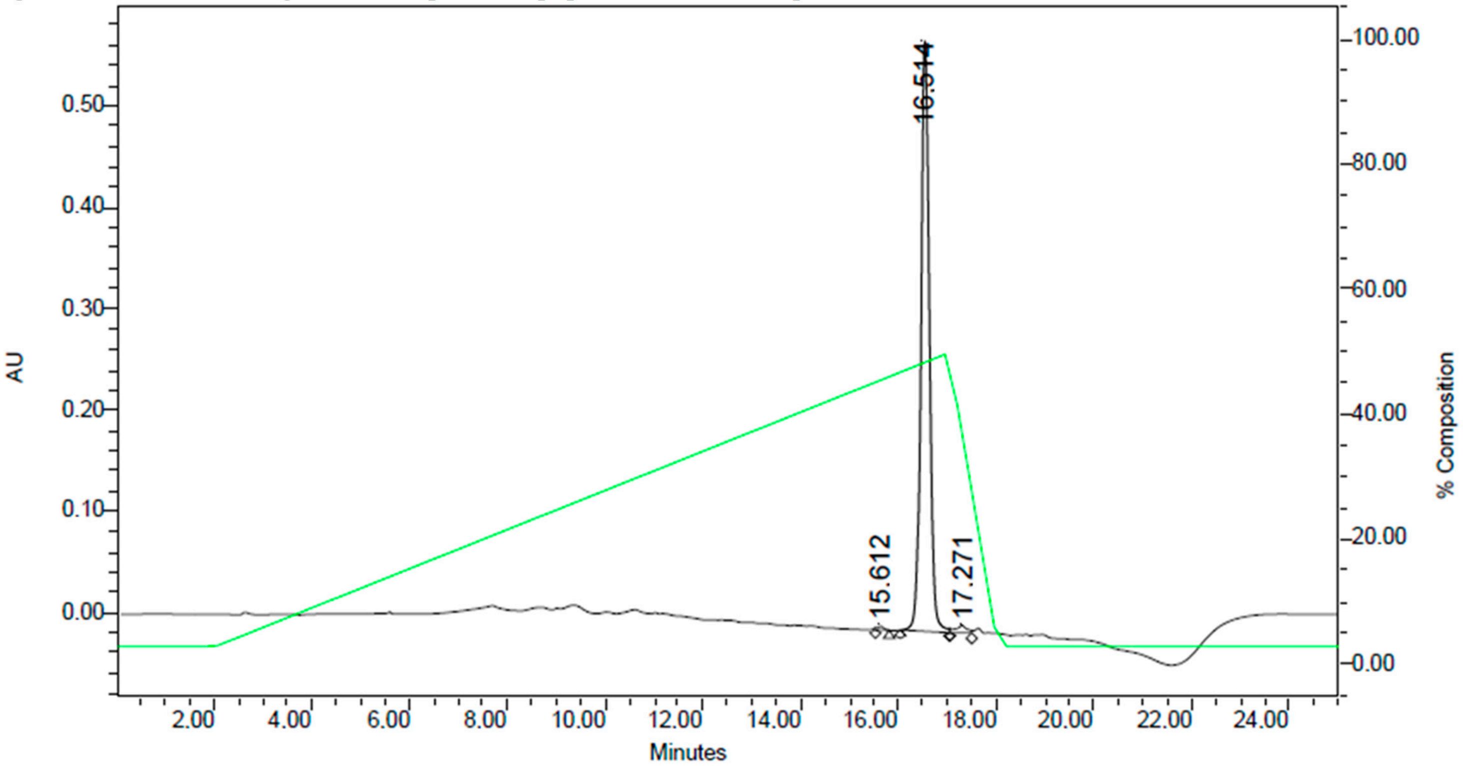

Figure S3. The chromatogram of sample R23DI peptide (run time for peak of R23DI was 16.514 min).

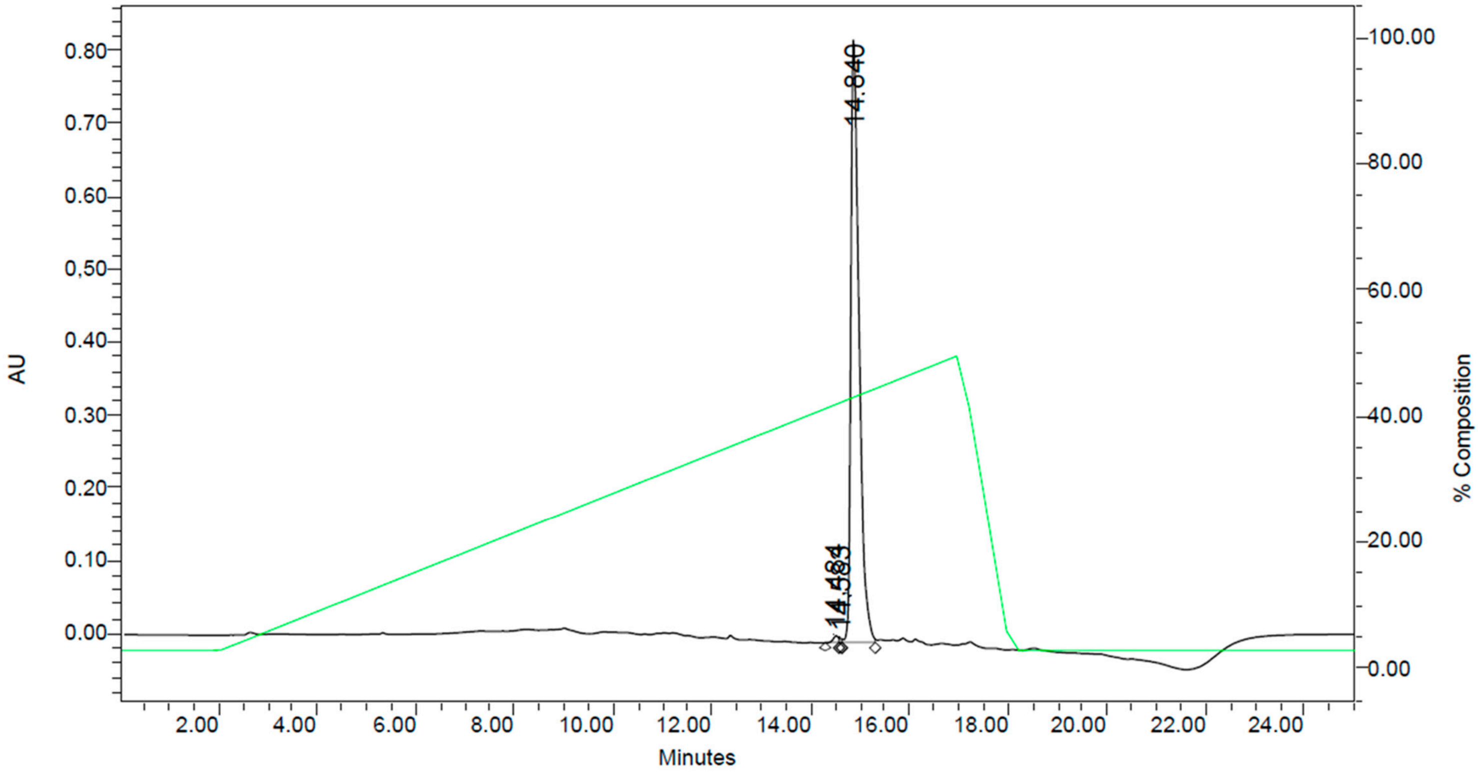

Figure S4. The chromatogram of sample R23EI peptide (run time for peak of R23DI was 14.840 min).

References

1. Conchillo-Solé, O.; de Groot, N.S.; Avilés, F.X.; Vendrell, J.; Daura, X.; Ventura, S. AGGRESCAN: a server for the prediction and evaluation of "hot spots" of aggregation in polypeptides. *BMC Bioinformatics* **2007**, *8*, 65, doi:10.1186/1471-2105-8-65.
2. Garbuzynskiy, S.O.; Lobanov, M.Y.; Galzitskaya, O. V. FoldAmyloid: a method of prediction of amyloidogenic regions from protein sequence. *Bioinformatics* **2010**, *26*, 326–332, doi:10.1093/bioinformatics/btp691.
3. Oliveberg, M. Waltz, an exciting new move in amyloid prediction. *Nat. Methods* **2010**, *7*, 187–188, doi:10.1038/nmeth0310-187.
4. Emily, M.; Talvas, A.; Delamarche, C. MetAmyl: A METa-Predictor for AMYLoid Proteins. *PLoS One* **2013**, *8*, e79722, doi:10.1371/journal.pone.0079722.
5. Wilkins, M.R.; Gasteiger, E.; Bairoch, A.; Sanchez, J.C.; Williams, K.L.; Appel, R.D.; Hochstrasser, D.F. Protein identification and analysis tools in the ExPASy server. *Methods Mol. Biol.* **1999**, *112*, 531–52, doi:10.1385/1-59259-584-7:531.
6. Pirtskhalava, M.; Amstrong, A.A.; Grigolava, M.; Chubinidze, M.; Alimbarashvili, E.; Vishnepolsky, B.; Gabrielian, A.; Rosenthal, A.; Hurt, D.E.; Tartakovsky, M. DBAASP v3: database of antimicrobial/cytotoxic activity and structure of peptides as a resource for development of new therapeutics. *Nucleic Acids Res.* **2021**, *49*, D288–D297, doi:10.1093/nar/gkaa991.
7. Waghu, F.H.; Barai, R.S.; Gurung, P.; Idicula-Thomas, S. CAMP R3 : a database on sequences, structures and signatures of antimicrobial peptides. *Nucleic Acids Res.* **2016**, *44*, D1094–D1097, doi:10.1093/nar/gkv1051.
